# A Comparative Review: Biological Safety and Sustainability of Metal Nanomaterials Without and with Machine Learning Assistance

**DOI:** 10.3390/mi16010015

**Published:** 2024-12-26

**Authors:** Na Xiao, Yonghui Li, Peiyan Sun, Peihua Zhu, Hongyan Wang, Yin Wu, Mingyu Bai, Ansheng Li, Wuyi Ming

**Affiliations:** 1 Department of Engineering, Huanghe University of Science and Technology, Zhengzhou 450008, China; 201608156@hhstu.edu.cn; 2 Henan Key Lab of Intelligent Manufacturing of Mechanical Equipment, Zhengzhou University of Light Industry, Zhengzhou 450002, China; yonghuili24@163.com (Y.L.); peiyansun252@163.com (P.S.); zphnszbd@163.com (P.Z.); hongyanwang923@163.com (H.W.); wuyin577@163.com (Y.W.); 3 Guangdong HUST Industrial Technology Research Institute, Huazhong University of Science and Technology, Dongguan 523808, China; wl1291242215@163.com; 4 Institute of Mechanical and Electronic Engineering, Henan Vocational College of Water Conservancy and Environment, Zhengzhou 450008, China

**Keywords:** metal nanomaterials, nanotoxicology, safety, sustainability, energy, environment

## Abstract

In recent years, metal nanomaterials and nanoproducts have been developed intensively, and they are now widely applied across various sectors, including energy, aerospace, agriculture, industry, and biomedicine. However, nanomaterials have been identified as potentially toxic, with the toxicity of metal nanoparticles posing significant risks to both human health and the environment. Therefore, the toxicological risk assessment of metal nanomaterials is essential to identify and mitigate potential adverse effects. This review provides a comprehensive analysis of the safety and sustainability of metallic nanoparticles (such as Au NPs, Ag NPs, etc.) in key domains such as medicine, energy, and environmental protection. Using a dual-perspective analysis approach, it highlights the unique advantages of machine learning in data processing, predictive modeling, and optimization. At the same time, it underscores the importance of traditional methods, particularly their ability to offer greater interpretability and more intuitive results in specific contexts. Finally, a comparative analysis of traditional methods and machine learning techniques for detecting the toxicity of metal nanomaterials is presented, emphasizing the key challenges that need to be addressed in future research.

## 1. Introduction

With the rapid development of science and technology, metallic nanomaterials have garnered significant attention due to their unique physicochemical properties and wide-ranging applications [1]. They have shown great potential in various fields, such as biomedicine, energy science, and environmental protection [2,3,4]. However, this broad applicability raises increasing concerns about their biosafety and sustainability, making biocompatibility and potential toxicity the focal points of research [5,6]. Additionally, the efficient and environmentally friendly preparation and application of these materials poses challenges from a sustainability perspective.

Over the past decades, studies on the biosafety and sustainability of metallic nanomaterials have primarily relied on traditional experimental methods and theoretical analyses [7,8,9]. While these methods provide valuable data and insights, they are often time-consuming, labor-intensive, and insufficient for comprehensively assessing the complex properties and behaviors of the materials. Recently, artificial intelligence (AI) technology has been extensively utilized across various fields to address complex challenges [10,11]. Machine learning (ML) is a subcategory of AI that uses algorithms to identify patterns in data and make predictions [12]. ML relies on various algorithms to address data problems, and the type of algorithm employed hinges on factors such as the nature of the problem you wish to solve, the number of variables involved, and the most suitable model type. The following are some commonly used algorithms in ML: Support Vector Machine (SVM), Artificial Neural Networks (ANNs), Random Forest (RF), Genetic Algorithm (GA) [13]. The ML approach has found widespread application in various industries, including printed circuit boards [14], hydrological forecasting [15], fuel cells [16,17], and other industries [18]. ML algorithms can analyze large datasets, predict, and optimize material properties [19]. In the context of metallic nanomaterials, ML can efficiently analyze experimental data, offering new perspectives and methods to understand and improve material properties. The biosafety of metallic nanomaterials is complex and multidimensional [20], influenced by chemical composition, size, shape, surface properties, environmental conditions, exposure time, and biological systems [21]. Traditional methods, such as cellular and animal experiments, are intuitive and effective but limited by experimental conditions, sample size, and time costs. Furthermore, due to the complexity of biological systems, experimental results are sometimes difficult to generalize to humans or broader ecological environments [22]. In contrast, ML techniques can extract useful information from large datasets and construct predictive models to assess biosafety. By identifying key factors associated with toxicity, ML can enhance prediction accuracy and accelerate the screening and optimization of metallic nanomaterials [23]. Additionally, ML can elucidate the interaction mechanisms between materials and biological systems, guiding the design of safer and more biocompatible nanomaterials [24].

Regarding sustainability, the preparation and application of metallic nanomaterials involve resource consumption, energy use, and environmental pollution [25]. Traditional sustainability assessment methods, such as life cycle assessment (LCA), are often limited by data acquisition and processing capabilities [26]. ML offers a more efficient and comprehensive approach to sustainability assessment. By analyzing extensive data, ML can identify key environmental impact factors, optimize resource use, and reduce energy consumption and environmental pollution [27,28]. The application of ML in the study of biosafety and sustainability of metallic nanomaterials is still in its infancy, with current research focusing on data collection, processing, and model optimization [27]. As data availability increases and algorithms improve, ML is expected to play a more significant role in these areas.

In summary, this overview systematically compares methods with and without machine learning assistance in the context of metal nanomaterials safety and sustainability research. The dual-perspective analysis highlights the distinct advantages of ML in data processing, predictive modeling, and optimization. Concurrently, the importance of traditional methods is examined, particularly their capacity to provide more explanatory and intuitive results in specific scenarios. This review offers new insights and a forward-looking discussion on metal nanomaterials safety and sustainability, contributing to the existing literature and potentially advancing the field.

## 2. Toxicity of Metal Nanomaterials

**Figure 1:**
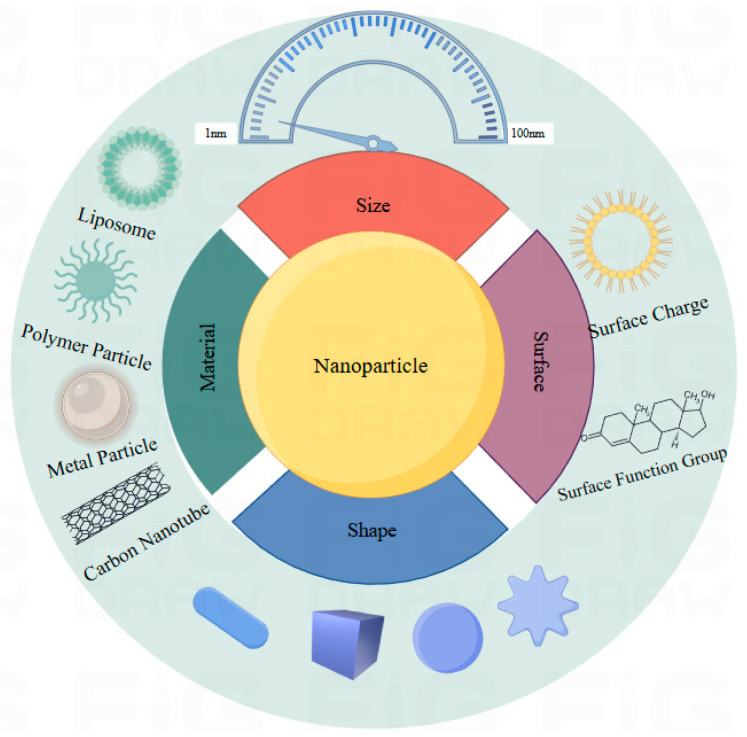
NPs’ composition, size, shape, and surface are related to their properties and applications.

Although there is great potential in the application of nanotechnology in various fields, analysis of the risks and drawbacks of using nanotechnology is necessary [29]. A branch of biological nanoscience called nanotoxicology is used to evaluate the toxic effects of nanomaterials on living things and other biological systems [30,31]. Behavior and toxicity are influenced by their physical and chemical properties, including their size, shape, and surface properties such as surface area, surface charge, solubility, and catalytic activity [32,33], as shown in Figure 1. Because of their small size, NPs can infiltrate living things and cause cell harm by piercing biological barriers like cell membranes. Additionally, when the size of a nanomaterial decreases, its surface-to-volume ratio increases substantially. The surface of NPs can then hold more chemical components, increasing their reactivity and harmful consequences.

Uptake is an important way that humans to come into contact with nanomaterials, including direct and indirect contact. Direct contact with the human body occurs by the dissolution of food or nanoparticles in food containers, while indirect contact occurs through secondary uptake and the inhalation of nanoparticles [34]. After entering the body, they may spread throughout it by way of blood circulation. Size and surface properties such as polarity, hydrophilicity, lipophilicity, and catalytic activity may influence their distribution in vivo [35,36]. Another significant way that human bodies are exposed to nanoparticles in the air is through inhalation [37]. Skin absorption is another important way for humans to come into contact with nanomaterials [38]. Because of their small size, NPs are easier for cells to take up and move between endothelium and epithelial cells. NPs can interact with tissues in different parts of the body to extend their stay there. The liver, kidney, and spleen are the primary organs where NPs are concentrated. They also have harmful effects on the lungs, heart, kidney, skin, and intracellularly [39]. Therefore, precautions should be taken to carry out a separate assessment of the potential dangers to human health and the environment of the use of novel nanomaterials.

Metal nanomaterials have the potential to reduce waste generation and industrial pollution, as well as improve energy production and utilization efficiency [40]. Nevertheless, the creation, application, and disposal of produced NPs will release pollutants into the air, the land, and the water supply [41], and increased public exposure to metal nanomaterials and their adverse health effects are urgent concerns. Yet it is anticipated that the toxicity of metal nanomaterials would depend on their particular size, shape, morphology, composition, distribution, dispersion, surface area, surface chemistry, and reactivity [42], complicating their toxicity assessment [43]. Currently, there is no globally accepted toxicity test or validation technique for the evaluation of metal nanomaterials’ safety [44]. Therefore, toxicological testing methods for metal nanomaterials will advance knowledge of their possible negative impacts on human health. Only with a detailed understanding of the toxicity of materials under different conditions and different environments can they be reasonably and safely used in various fields.

## 3. Medical Field

A significant class of nanoparticles that have been created due to their properties as thermoelectric, electroluminescent, and semiconductor materials are metal nanomaterials [45]. In drug delivery systems, these antimicrobial nanoparticles have been employed to enter areas of biology that are inaccessible through conventional medications. Numerous studies have been carried out to determine whether the original properties of these nanomaterials, such as high surface area to volume ratios, have a negative environmental impact in light of the recent growth in the interest and development in nanotechnology [46]. Since then, researchers have identified numerous deleterious effects of various metals and metal oxide nanomaterials on cells. For instance, DNA oxidation and fragmentation, mutations, morphological alterations, decreased cell viability, apoptosis, necrosis, and decreased proliferation were all factors [47].

### 3.1. Traditional Method in Medical Field

**Figure 2:**
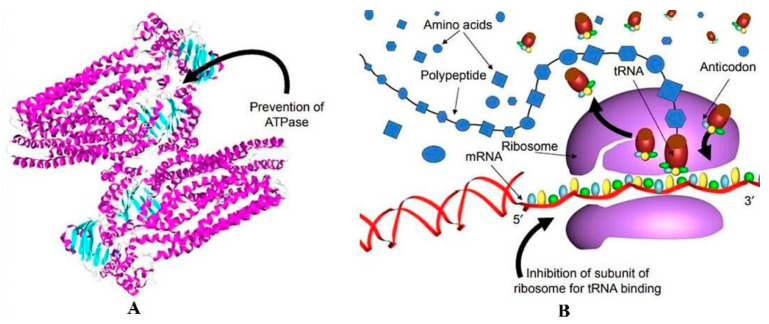
Two antimicrobial mechanisms of gold nanoparticles: (**A**) alteration of membrane potential and inhibition of ATPase activity. (**B**) Inhibition of tRNA-binding components of ribosomes [49].

**Au NPs:** Gold nanomaterials (Au NPs) are fast, simple, and inherently bioinert, which make them excellent candidates for biomedical applications because of their good bio-coupling potential [48]. Although the antimicrobial mechanism of Au NPs is not as well studied, it is believed that the antimicrobial activity of Au NPs mainly occurs through the following two mechanisms: (1) The shift in membrane potential and the suppression of ATPase activity contribute to the decrease in cell metabolism (Figure 2A). (2) The ribosomal subunit required for tRNA binding is inhibited, which results in the breakdown of biological processes (Figure 2B) [49]. The interaction of Au NPs with biological systems, namely cytotoxicity, must be thoroughly studied because they are the most widely utilized metal nanoparticles in the biomedical industry. This is necessary to determine their long-term impact on human health. Moore et al. [50] observed that cellular uptake of Au NPs and DNA binding via gene therapy affects the size and shape of plasmid DNA. Semmler-Behnke et al. [51] employed an in vivo experimental approach within animals to conclusively demonstrate, for the very first time, that extremely minute gold nanoparticles (1.4 nm in diameter) possess the remarkable ability to traverse the air/blood barrier of the respiratory tracts in substantial amounts, forming a stark contrast to 18 nm particles, which are almost exclusively retained within the confines of the lungs. Zhang et al. [47] explored in vivo experimental endeavors within animals and uncovered a pivotal finding: gold nanoparticles and nanoclusters exhibited toxicity within the biological milieu, leading to a reduction in the count of red blood cells and inflicting damage upon vital organs such as the spleen, liver, and kidney. Zheng et al. [52] demonstrated, through direct microscopic observation, that reducing the size of gold nanoparticles to the nanocluster range (2 nm in diameter) significantly enhanced their antimicrobial activity against both Gram-positive and Gram-negative bacteria by elevating intracellular reactive oxygen species (ROS) levels, without imposing additional cytotoxic or genotoxic burdens on host cells. Bin-jumah et al. [53] synthesized green gold nanoparticles using leaf extract of *Azadirachta indica* and auric chloride, and studied their cytotoxic, genotoxic, and apoptotic effects on human normal (CHANG) cells and hepatocellular carcinoma (HUH-7) cells. The study revealed that green Au NPs induced cytotoxic and apoptotic changes in both HUH-7 and CHANG cells. Notably, green Au NPs were found to be more harmful to healthy cells than to liver cancer cells, with oxidative stress identified as the main factor contributing to this toxicity.

**Figure 3:**
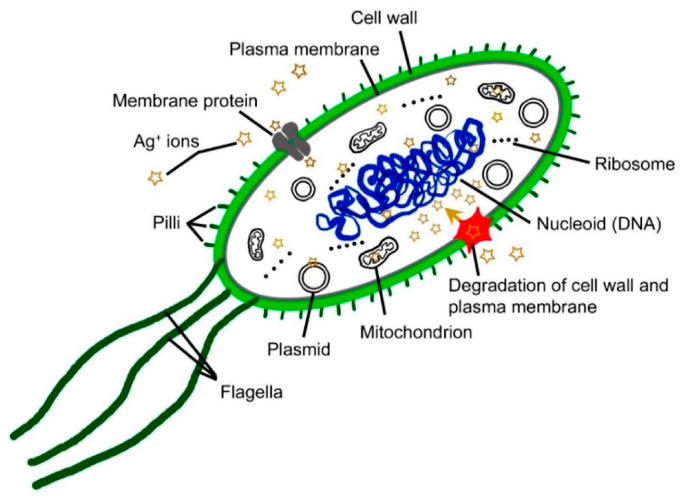
Multifaceted mechanisms of the antibacterial action of silver ions [55].

**Ag NPs:** Silver nanoparticles (Ag NPs) are particles ranging in size from 1 to 100 nm, typically consisting of 20 to 15,000 silver atoms [54]. Silver demonstrates amazing physical, chemical, and biological qualities at the nanoscale. Because of its powerful antimicrobial activity (Figure 3), numerous textiles use nano-silver coatings, as well as it also being in the coating of certain implants [55]. Additionally, Ag NPs are sold as water disinfectants and room sprays and are used to heal burns, wounds, and as a contraceptive [56]. With the increasing use of Ag NPs in medical and related applications, there are also increasingly serious toxicological and environmental issues. Ag NPs can be exposed through oral delivery, although it is unclear how they work during gastrointestinal digestion [57]. Hussain et al. [58] evaluated the potential toxicity of silver nanoparticles through a study method that involved exposing rat liver-derived cells (BRL 3A) to silver nanoparticles in vitro. The toxicity was laterally assessed by examining changes in cell morphology, mitochondrial function (using the MTT assay), membrane leakage of lactate dehydrogenase (LDH assay), reduced glutathione (GSH) levels, and reactive oxygen species (ROS) production post-exposure. The study revealed that silver nanoparticles exhibited high toxicity, with a notable decrease in mitochondrial function and an increase in LDH leakage in rat liver-derived cells exposed to 5–50 μg/mL of Ag Nps. Li et al. [59] utilized the Salmonella reverse mutation assay (Ames test) and an in vitro micronucleus assay to evaluate the genotoxicity of silver nanoparticles. Human lymphoblastoid TK6 cells were treated with 10–30 μg/mL of Ag NPs, while other cells served as controls, treated with water and exposed to 0.73 gy of X-rays as positive controls. The findings indicated that Ag NPs induced a significant 3.17-fold increase in the vector control and a net 1.60% increase in micronucleus frequency at a concentration of 30 μg/mL Ag NPs, accompanied by a 45.4% increase in the relative population. These results suggest that 5 nm Ag NPs are genotoxic in TK6cells. Mirsattari et al. [60] reported a case of a 71-year-old male patient who consumed colloidal silver every day for four months before experiencing myoclonic status epileptoid, coma, and elevated silver levels in his blood, red blood cells, and cerebrospinal fluid. High quantities of silver have been found in plasma, red blood cells, cerebrospinal fluid, in addition to myoclonic convulsions in individuals after long-term chronic colloidal silver intake.

**Figure 4:**
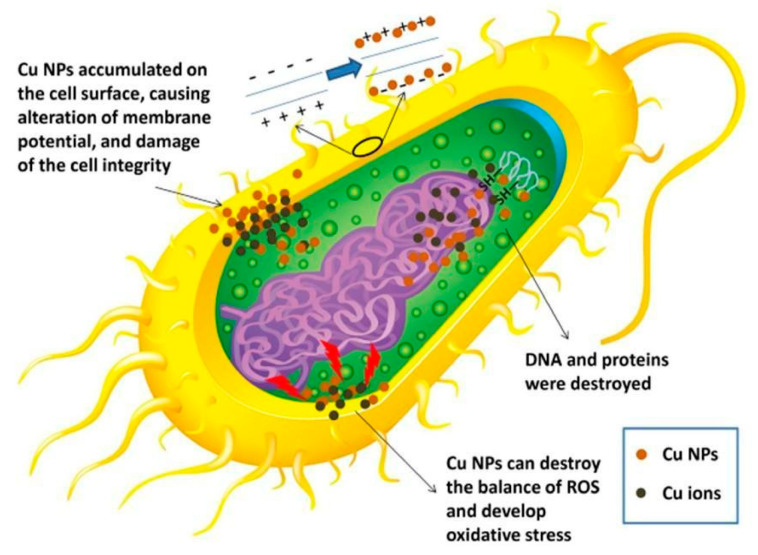
The antibacterial mechanism of copper nanoparticles [63].

**Cu NPs**: Fungi have become a substantial menace to human health in recent years, so it is urgent to develop advanced coating technology for application in biomedical devices or antibacterial packaging. Metal/polymer nanocomposites are an effective choice for this purpose, and copper nanoparticles (Cu NPs), as a highly effective antibacterial agent, are the preferred research object [61]. Cu NPs demonstrated strong antibacterial action against Methicillin-resistant *Staphylococcus aureus* (MRSA), equivalent to Ag NPs and certain drugs in terms of bactericidal efficacy. Cu NPs’ antibacterial action involves the binding of released copper ions to DNA, just like Ag does [62], as shown in Figure 4 [63]. Compared with silver and gold nanoparticles, there are few studies on the cytotoxicity of Cu NPs [64]. According to Chen et al. [65], if a person consumes more copper than their body can tolerate, it can cause toxic symptoms such as hemolysis and jaundice, which can ultimately result in death. Cu NPs may simultaneously result in pathological damage to the spleen, kidney, and liver. In addition, Yang et al. [66] discovered that Cu NPs were hazardous to the brain. Prabhu et al. [67], through their in vitro experimental approach utilizing animal cell cultures, revealed that Cu NPs exhibited a pronounced toxic effect on dorsal root ganglion (DRG) neurons at concentrations ranging from 40 to 100 μm, whereas at lower concentrations of 10–20 μm, they did not significantly impair the viability of these neurons. Notably, the study underscored that smaller Cu NP sizes and elevated concentrations tend to exacerbate their toxic potential. Xu et al. [68] employed a methodology to evaluate toxicity by assessing the dose–response relationship of nanoparticles within cells, investigating the cytotoxic effects of Cu NPs on PC12 rat pheochromocytoma cells, which were widely used as an in vitro model for the neuron research. Their findings revealed that exposure to Cu NPs induced oxidative stress, manifested as increased ROS levels, suppressed superoxide dismutase (SOD) activity, and ultimately led to cell death, all in a concentration- and time-dependent manner.

### 3.2. Machine Learning Methods in Medical Field

The application of ML techniques to metal-based nanomaterials has significantly advanced the understanding of nanoparticle interactions, property prediction, and the discovery of new materials. However, the toxicity of nanomaterials cannot be overlooked, as different metal-based nanomaterials can have varying harmful effects on human health and the environment. Given the high dimensionality and nonlinear nature of datasets in the study of metal-based nanomaterials, along with the complexity of nanotoxicity issues, recent advances in ML techniques are particularly important for addressing these challenges.

**Figure 5:**
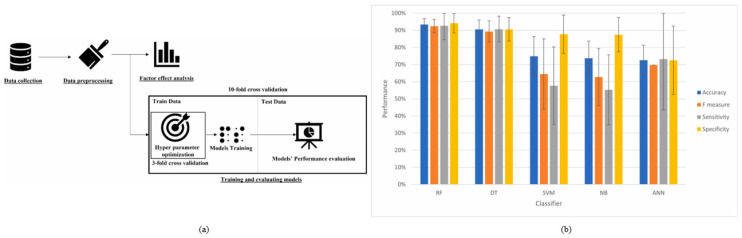
Flow of ML algorithms and model performance. (**a**) Experimental workflow for ML to predict nanoparticle toxicity. (**b**) Performance comparison of five ML models [69].

**Au NPs:** Ahmadi et al. [69] used data mining to evaluate and model factors affecting the toxicity of Au NPs. The experimental workflow for ML used to predict nanoparticle toxicity is shown in Figure 5a. In this study, five data mining models—Decision Tree (DT), RF, SVM, Naïve Bayes (NB), and ANN—were employed to predict the toxicity of nanoparticles. A comparison of the models’ performance is illustrated in Figure 5b. From Figure 5b, it can be observed that the RF model achieves the highest performance metrics, with an accuracy of 93.45%, an F-measure of 92.44%, sensitivity of 92.70%, and specificity of 94.18%. Predicting the cytotoxicity of nanoparticles is a common application of data mining and ML in nanoinformatics research [70]. Various prediction methods, including multiple regression, logistic regression, and Monte Carlo methods, are employed to determine the cytotoxicity of gold nanoparticles. In a study by Winkler et al. [71], Bayesian neural networks and multiple linear regression models were used to determine protein binding to the surface of Au NPs. Winkler et al. also predicted the effect of nanoparticle uptake by human pancreatic ductal adenocarcinoma cells and human umbilical vein endothelial cells lines using nonlinear Quantitative Structure–Activity Relationship (QSAR) models. These simulations suggest that multiple interactions between nanoparticles and cells may cause cytotoxicity. Factors such as size, shape, dosage, aggregation state, surface charge, and chemistry are crucial in understanding cytotoxicity [72,73]. Lin et al. [74] found that larger nanoparticles resist stochastic force fluctuations, while smaller particles move rapidly, leading to increased aggregation. Smaller gold nanoparticles are more likely to cause toxicity due to their higher surface area relative to mass, increasing their interactions with biomolecules. Additionally, ions affect the physical and chemical properties of nanoparticles. 

**Figure 6:**
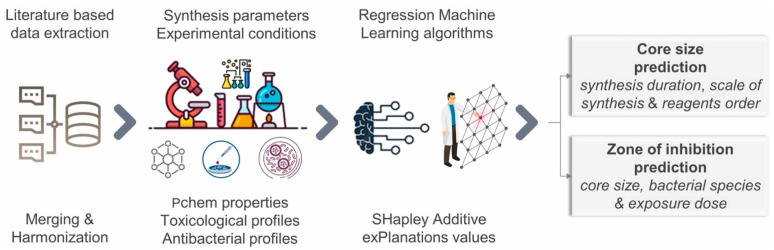
Prediction of antimicrobial efficiency of Ag NPs using ML algorithms [76].

**Ag NPs:** Due to their complexity and multidimensionality, traditional methods struggle to systematically explore the entire parameter space and understand the relationship between experimental synthesis conditions and the toxicity of Ag NPs. Additionally, manual experiments are time-consuming, expensive, and limited in scope, making it difficult to efficiently optimize synthesis conditions. Therefore, modeling techniques such as ML models offer a promising approach to harness this complexity and capture the interactions of multiple parameters. Liu et al. [75] used two classification-based ML methods, DT and RF, to meta-analyze the cytotoxicity data of plant-synthesized Ag NPs with heterogeneous profiles from the literature. The experimental results showed that the accuracy and generalization performance of DT and RF models were significantly improved, with RF achieving the highest accuracy (0.825) and predictive ability (AUC = 0.904). The potential influence of biosynthetic parameters on the cytotoxic effects of plant-synthesized Ag NPs was revealed. Furxhi et al. [76] manually extracted data related to synthesis from the literature and used PyCaret regression library to train various ML algorithms (Linear regression, Decision Tree regression, Random Forest regression, and Support Vector regression, etc.). They used regression algorithms to establish the relationship between the synthesized parameters and the expected endpoints, which led to a predictive model. The core size and antimicrobial efficiency models were trained and validated using cross-validation methods. The whole experiment is shown in Figure 6. The regression algorithms performed similarly with Random Forest and Extreme Gradient Boosting, reaching during the 10-fold cross a R^2^ ≈ 0.70, root mean square error (RMSE) of 16.85, and average absolute error (MAE) of 9.10. Desai et al. [77] investigated the relationship between the toxicity of Ag NPs and physical input features such as reductant, particle size, zeta potential, cell type (cancer/normal cell line), hydrodynamic diameter, wavelength, morphology, exposure time, and dose through ML. Supervised ML algorithms, using DT and RF for regression analyses, were employed in their study. The test scores obtained showed perfect accuracy with DT ($R^{2}$ = 1) compared to RF. The results indicated that the high $R^{2}$ values and low RMSE values demonstrating that DT outperforms RF in predicting toxicity parameters and is best suited to the datasets.

**Others:** Kovalishyn et al. [78] compiled data from the literature on the toxicity of metal and metal oxide nanoparticles to various organisms and uploaded it to the online chemical modeling environment database. Key characteristics of the nanoparticles, such as chemical composition, average particle size, shape, surface charge, and bioassay species information, were used as descriptors for developing a QSAR model. The QSAR methodology utilized Random Forests (WEKA-RF), k-nearest neighbors, and associative neural networks. The predictive power of these models was evaluated through cross-validation, with cross-validation coefficients (q^2^) ranging from 0.58 to 0.80 for regression models and equilibrium accuracies between 65% and 88% for classification models. Varsou et al. [79] proposed an automated machine learning (auto ML) scheme using silver (Ag), titanium dioxide (${\mathrm{TiO}}_{2}$), and copper oxide (CuO) nanoparticles for dose–response toxicity data. This model is based on dose–response toxicity data, with calculated atomic descriptors of nanoparticles used as independent variables to predict the toxicity class (low or high toxicity) of the nanoparticles. High toxicity data were oversampled to balance category representation by generating synthetic data using Synthetic Minority Over-sampling Technique (SMOTE) and Adaptive Synthetic Sampling Approach for Imbalanced Learning (ADASYN) methods. The quality of the synthetic data was assessed to ensure compatibility with the original data.

### 3.3. Summary

This section provides an in-depth discussion of the toxicity prediction of metal nanoparticles in the medical field. Table 1 compares the differences between traditional and ML methods for detecting the toxicity of metal nanomaterials in the medical field.

## 4. Energy Field

Metal nanoparticles have been widely used in the field of energy because of their high surface area, high selectivity, tunable morphology, and significant catalytic activity [80]. The working principle of catalysts is to reduce the activation energy of biochemical reactions. The problem of how to improve the efficiency of catalysts also appears in front of people in order to improve working efficiency and maximize economic benefits. Compared with the traditional oxide catalyst, the metal nanoparticle catalyst has more active sites, so its catalytic efficiency is greatly improved [81]. Chemically, the structure and morphology of metal nanoparticles can be changed, and the combination of different metal nanoparticles can greatly improve the catalytic efficiency. However, studies have shown that some metal nanoparticles used for energy catalysis are toxic under specific conditions, and their toxicity depends on various factors.

### 4.1. Traditional Method in Energy Field

**Ag NPs:** Ag NPs are employed in a variety of sophisticated catalytic technologies as well as nanomedicine because of their distinctive qualities and traits. The extensive advantages of catalytic activity, selectivity, stability, and recovery are what give the synthesis and use of Ag NPs their scientific relevance. Therefore, a new generation of chemists will be motivated by the growth and development of Ag NPs catalysts to customize and create potent catalysts that will assist in solving significant environmental problems and displace precious metals in sophisticated catalytic applications [82]. However, frequent usage of Ag NPs considerably increases the likelihood of human interaction. Moreover, a large number of in vitro studies have shown that Ag NPs will damage the skin, various organs, and blood vessels of mammals and will also harm the survival of animals and plants when it enters the environment [83]. Lam et al. [84] used dermal fibroblasts as the feeder layer to study the toxicity of nanocrystalline silver dressing (Acticoat) in the culture medium. The experimental results showed that Acticoat had serious cytotoxic effects on keratinocytes and should not be used as a local dressing for cultured skin grafts. Arora et al. [85] studied the in vitro interaction of Ag NPs with primary fibroblasts and primary hepatocytes from Swiss albino mice and demonstrated that Ag NPs can enter cells. This causes DNA damage and apoptosis in fibroblasts and hepatocytes and induces death and oxidative stress in human fibrosarcoma and skin cancer cells. In their study, the mouse cells were subjected to Ag NP solution at concentrations of 30 μg/mL and 225 μg/mL, respectively, to observe the biochemical changes in the cells. Hsin [86] concluded that Ag NPs were cytotoxic and could induce the apoptosis of NIH3T3 fibroblasts. The apoptosis induced by Ag NPs was related to the generation of ROS and the activation of JNK (c-Jun N-terminal kinase), indicating that Ag NPs induced cell apoptosis through the mitochondrial pathway through ROS and JNK. After examining the Ag NP content in various mouse organs following exposure using morphology and inductively coupled plasma mass spectrometry (ICP-MS) analysis, Takenaka et al. [87] came to the conclusion that the nasal cavity, particularly the posterior part, and the lung-related lymph nodes displayed relatively high concentrations of Ag.

**Pt NPs:** Due to platinum nanoparticles (Pt NPs)’ great chemical stability and catalytic activity, they play a significant role in the energy sector, particularly in catalysis [88]. In air and water, platinum’s chemistry is inert and stable, and it is simple to form complexes and certain intermediates that have higher activity. As a result, platinum is one of the most significant catalytic materials. As early as 1831, platinum was effectively utilized as a catalyst for the manufacture of sulfuric acid. Since then, platinum-based catalysts have drawn an increasing number of researchers due to their superior selectivity, stability, and catalytic activity. With the increasing popularity of Pt NPs for energy use, some researchers have found that Pt NPs can be cytotoxic in some specific situations. Madlum et al. [89] prepared high-purity Pt NPs of two sizes (10 nm and 20 nm) for the evaluation of bioactivity using three pathogenic microorganisms, *Pseudomonas aeruginosa*, *Staphylococcus aureus*, *Escherichia coli*, and *Candida albicans*, and two cell lines (Hepa 1–6), hepatocellular carcinoma cells and MDCK cells, and then conducted antimicrobial experiments on these particles. The results of the MTT assay showed that Pt NPs with particle sizes of 10 nm had higher toxicity than Pt NPs with particle sizes of 20 nm at the same concentration, and low concentrations of Pt NPs had a higher cytotoxic effect on cancer cells. In two membrane-emerging trophoblast (EVT) cell lines, Nakashima et al. [90] investigated the activation of autophagy by 1 nm-sized Pt NPs. Experimental results demonstrate that Pt NPs can activate autophagic flux in both cell lines. Additionally, Pt NPs impair the functionality of EVT cell lines and inhibit their proliferation. However, the autophagosomes generated by the activation of autophagic flux induced by Pt NPs themselves can mitigate the cytotoxic effects of Pt NPs.

**Ir NPs:** Iridium (Ir) is chemically stable and necessary for many chemical processes. It often occurs in nature as a natural element or alloy. Its catalytic activity was discovered by Lauri Vaska in 1960 [91]. In both alkaline and acidic media, it is the most potent and often utilized catalyst for many processes [92,93]. Ir and its complexes are used as catalysts in nano-, bio-, and homogeneous processes. It is the most fundamental element in the platinum group and has the widest range of oxidation positions of all transition elements, ranging from −3 to +9. Ir is used in a variety of applications, such as catalysis, medicine, and manufacturing, as a result of these facts [94,95,96]. During photosynthetic processes, it is in charge of breaking water molecules into hydrogen and oxygen [97]. Ir could therefore solve the food crisis through artificial photosynthesis, as well as the energy crisis by breaking down water to produce hydrogen fuel (green energy). Although Ir nanoparticles are promising in a variety of applications, there are still some safety-related issues. Buckley et al. [98] exposed rats’ nasal cavities to radioactive Ir-192 nanoparticle aerosol produced by sparks for three hours. The particle sizes were 10 nm, 15 nm, 35 nm, and 75 nm (median diameter of counting) (aerosol mass concentrations were 17, 140, 430, and 690 μg/$m^{3}$, respectively). Ir-192 was measured in whole animals, organs, tissues, and excreta at different times, 1 to 2 months after exposure. The results indicate that brain displacement was below the detection threshold (i.e., less than 0.01% of the lung content). The pulmonary clearance of inhaled inert aerosol particles smaller than 100 nanometers was slow, with a transient increase in concentration noted in the primary secondary target organs. These findings may be linked to chronic toxicity, particularly with prolonged exposure.

### 4.2. Machine Learning Methods in Energy Field

**Figure 7:**
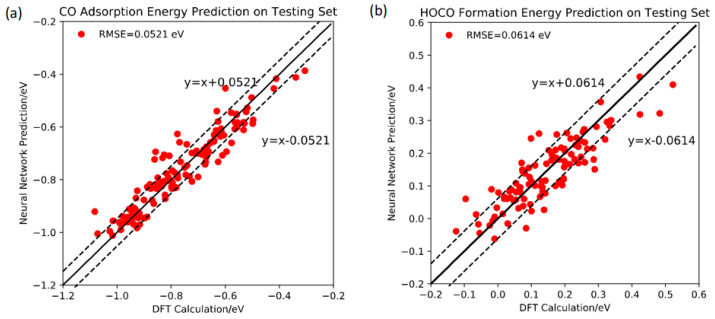
Predictive performance of ML models (**a**) ML model prediction of CO adsorption energy on the surface of Au NPs shows RMSE of 0.0521 eV. (**b**) ML model prediction of HOCO generation energy on the surface of Au NPs showing RMSE of 0.0614 eV [100].

**Au NPs:** Catalysts are crucial for key reactions such as the oxygen reduction reaction, hydrogen evolution reaction, and oxygen evolution reaction within the energy sector [99]. To better evaluate the sustainability of nanomaterials as efficient catalysts, numerous experiments have demonstrated that the surface of Au NPs significantly enhances the performance of carbon dioxide reduction to carbon monoxide (CO_2_RR). However, the underlying surface features responsible for this improved performance remain unexplained. To address this, Chen et al. [100] employed ML techniques, developing two neural network-based ML models to accurately predict the performance (a-value) of surface sites on Au NPs, as well as the CO adsorption energy and HOCO generation energy on highly irregular and disordered Au NP surfaces. The required accuracy for catalyst development was found to be 0.05 eV. The prediction of CO adsorption energy and HOCO generation energy on the Au NP surface by the ML models shown in Figure 7a,b can help in the design of high-performance electrocatalysts for CO_2_RR, solar energy storage, and CO_2_ transport. Additionally, in order to better understand the thermal stability of nanomaterials in their safety in the energy sector [101], Zeni et al. [102] developed a machine learning force field (MLFF) using a novel framework based on mapped Gaussian processes. They simulated the melting process of Au NPs containing 309 atoms and with a diameter of about 2 nm. They used two datasets to train the ML-FF model: one generated using the Revised Perdew–Burke–Ernzerhof (rPBE) functional and the other using the Local Density Approximation (LDA) functional. The MLFF exhibited an MAE in force components of 0.09 ± 0.04 eV/Å (LDA ML-FF) and 0.07 ± 0.03 eV/Å (rPBE ML-FF), while the MAE in atomic energy differences was 2.65 ± 2.02 meV/atom (LDA ML-FF) and 1.98 ± 1.76 meV/atom (rPBE ML-FF). Their experimental data indicated that the MAE for force components was around 0.1 eV/Å and the MAE for atomic energy differences was below 10 meV/atom. This study provides a highly accurate ML model for predicting the melting temperature of gold nanoparticles and analyzing their thermal stability, offering valuable insights for safety assessments in the energy sector.

**Ag NPs:** Over the past decade, nanofluids have garnered significant attention for their potential in enhancing heat transfer technologies and advancing renewable and sustainable energy systems. By incorporating nanoparticles into base fluids, nanofluids have demonstrated superior thermophysical properties, including improved heat transfer and increased energy absorption and storage, compared to the base fluids alone [103,104,105]. Therefore, investigating the thermophysical properties of nanofluids is crucial for evaluating the safety and sustainability of nanomaterials within the energy sector. Toghraie et al. [106] studied the dynamic viscosity of silver nanofluids across a temperature range of 25–55 °C and nanoparticle volume fractions ranging from 0.2% to 2%. They utilized an ANN to predict viscosity values and compared these predictions with laboratory measurements. The experimental data show that the MSE of the calculated method is 0.0012 and SSE is 0.0512 with a maximum error of 0.0858. The experimental results indicate that the synthesis of silver nanoparticles with controllable physicochemical properties is essential to optimize their functionality and safety. In recent years, nanoparticles have also been extensively used to enhance the efficiency of solar cells [107]. The sustainability analysis of nanoparticles can be initiated by examining their optical properties. Kashiwagi et al. [108] synthesized silver nanoplates using a high-throughput method and analyzed the synthesis process with ML tools, employing Python software (version 3.7) and the scikit-learn library (version 0.23.1). They applied a nonlinear regression model to compare the peak wavelengths of the absorption spectra of silver nanoplates with those predicted by the model. The study elucidated the effects of silver nitrate concentration, TSC concentration, and seed volume on the absorption spectra. This research provides optimal parameters for synthesizing nanoplates with desirable optical properties through a high-throughput approach combined with ML analysis.

**Others:** Tamtaji et al. [109] developed a general and linearly correlated descriptor by analyzing the significance of features derived from a neural network-based ML algorithm. Using this descriptor, they synthesized gold-silica nanoparticle catalysts and assessed their plasma strengths through scanning transmission electron microscopy-electron energy loss spectroscopy (STEM-EELS) mapping. Experimental results demonstrated that the photosynthetic reaction rate of anthracene increased three-fold and the photo-oxidative selectivity of dihydroartemisinic acid improved by 4% when θ = 0.4 for gold-silica nanoparticles, achieving a significant milestone in organic synthesis. Tamtaji et al. outlined the preparation of highly efficient gold-silica nanoparticle catalysts, which is crucial for nanoparticle sustainability analysis and the development of catalysts for organic drug synthesis. In the energy sector, hydrogen energy is a vital resource, and efficient energy storage technologies are increasingly important due to rising energy consumption [110]. The application of nanofluids in hydrogen storage systems enhances thermal management due to their superior heat transfer characteristics and stability [111]. Urunkar et al. [112] investigated the use of Al_2_O_3_/H_2_O, CuO/H_2_O, and MgO/H_2_O nanofluids as alternatives to traditional heat transfer fluids in a metal hydride reactor (MHR). They numerically modeled the hydrogen absorption process in an MmNi_4.6_Al_0.4_-filled MHR using ANSYS Fluent, based on various governing equations. The simulation results revealed that the CuO/H_2_O nanofluid increased the heat transfer rate by 10%, which in turn reduced hydrogen uptake time by 9.5%. The model was validated against published experimental data, showing deviations of less than 5%. This numerical model effectively predicts the heat transfer efficiency of metallic nanofluids and provides a robust evaluation of nanomaterials’ performance in various environments, enhancing their reliability and safety.

### 4.3. Summary

This section examines the biosafety and sustainability of metal nanoparticles in energy applications and provides an overview of the application of traditional and ML methods in this research, respectively. Table 2 contrasts traditional and ML approaches in evaluating the safety and sustainability of metallic nanomaterials in energy applications.

## 5. Environmental Engineering Field

Nanomaterials are widely regarded by researchers and scholars as one of the key pillars of emerging technologies. In the field of environmental engineering, high-performance metal nanomaterials are indispensable [113,114]. Soil and water pollution are pervasive environmental issues, and under normal conditions, pollutants are unlikely to naturally attenuate to safe levels. Specially synthesized metal nanoparticles have proven effective in remediating such pollution [115]. However, with the increasing application of metal nanoparticles in environmental engineering, their accumulation in the environment is expected to grow significantly, potentially posing substantial risks to human health and the environment [116].

### 5.1. Traditional Method in Environmental Engineering Field

**Figure 8:**
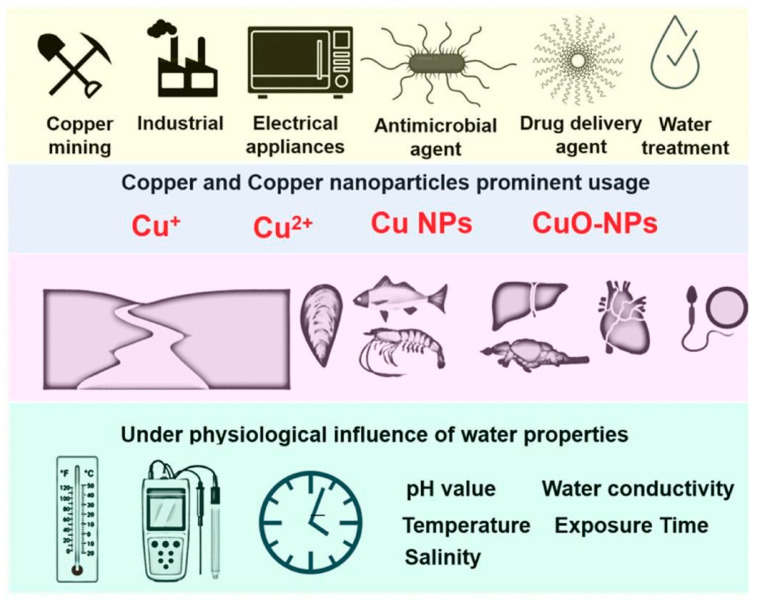
Schematic shows Cu and Cu NPs bioavailability, primary forms interacting with biota, and key toxicity factors in current and proposed applications [118].

**Cu NPs:** Copper and copper oxide particles are widely employed in various applications, including everyday products, maritime activities, drug delivery, paints, water treatment, and the agricultural and food industries. However, their extensive release into the environment poses significant risks to ecological systems and biological safety [117]. As depicted in Figure 8, these particles can ultimately enter human systems through trophic transfer. The yellow panel at the top illustrates potential sources of copper and Cu NPs pollution originating from daily activities. The central blue panel identifies various forms of copper contamination, such as Cu^+^, Cu_2_^+^, Cu NPs, and CuO NPs. The pink panel in the middle shows that aquatic organisms can ingest copper and Cu NPs, which then accumulate in internal organs, including the liver, brain, heart, and reproductive systems. The bottom green panel highlights the impact of environmental factors—such as pH, temperature, salinity, water hardness, and exposure duration—on the toxicity of copper and Cu NPs [118]. Kamunde et al. [119] utilized animal model testing to investigate the effects of varying concentrations of Cu NPs added to the diet of juvenile rainbow trout (*Oncorhynchus mykiss*). They assessed copper NP accumulation in liver and intestinal tissues over a 28-day period, noting the formation of numerous lamellar bodies, lysosomes, mitochondria, and other lamellar structures within intestinal epithelial cells. De Boeck et al. [120] explored the combined effects of elevated endogenous cortisol and sublethal copper exposure on freshwater carp. Their study found that exposure to Cu NPs resulted in significant damage, including disrupted anaerobic metabolism, gill damage, decreased plasma ion levels, and blood thickening.

**Ag NPs:** Ag NPs have been extensively studied for their environmental applications. For example, Ag NPs are often combined with cellulosic materials to monitor and address water contamination. These composites are valued for being cost-effective, readily available, environmentally safe, and recyclable. Various chemically functionalized Ag NPs can detect substances such as cadmium (II), cobalt (II), nickel (II), copper (II), mercury (II), lead (II), small organic molecules, diverse colors, pesticides, and antibiotics [121]. Colman et al. [122] demonstrated in their research on aquatic ecosystems that even Ag NPs coated with polyvinylpyrrolidone (PVP) and glutaraldehyde can exhibit toxicity. Their study observed a significant increase in methane content—up to 40 times higher than normal—as well as a notable decline in the density of healthy phytoplankton. In their experiment, gallic acid-coated Ag NPs (12 nm) and PVP-coated Ag NPs (49 nm) were introduced into a mesocosm at concentrations of 2.5 mg/L. Both types of Ag NPs were found to be toxic to aquatic plants, though PVP-coated Ag NPs were somewhat less harmful. The toxic effects included the release of dissolved organic carbon and chloride. These findings suggest that coating alone may not be sufficient to mitigate environmental impact.

### 5.2. Machine Learning Methods in Environmental Engineering Field

**Au NPs:** With the increasing integration of nanomaterials into society and industry, their annual production is rising rapidly, reaching approximately 242,000 tons annually, with 80% comprising metal or metal oxide nanomaterials [123]. As these nanomaterials are extensively applied in environmental contexts, they inevitably impact the environment. For instance, metal nanoparticles released into aquatic environments can affect various aquatic organisms, including fish, crustaceans, algae, and bacteria [124]. Investigating the effects of nanomaterials on aquatic organisms is crucial for assessing the safety and sustainability of nanoparticles in environmental applications. Zhou et al. [125] conducted a study on the eco-toxicity of metal nanomaterials towards aquatic organisms and developed a multi-species toxicity prediction model (ML-PEMST) utilizing ML techniques and environmental conditions. Additionally, three ML regression models—RF, SVM, and ANN—were employed to evaluate the ecotoxicity of Au NPs across different aquatic species. Among these, the Random Forest model achieved predictions with less than 10% deviation from experimental results, enhancing the interpretability of ML and uncovering complex feature interactions not easily detected by SVM or ANN. The study identified exposure time, light level, primary size, and hydrodynamic diameter as the principal factors influencing the ecotoxicity of Au NPs, with light level showing the most significant interactions with other variables. This research offers a novel approach for predicting nanoparticle ecotoxicity, contributing to a deeper understanding of their toxicity and sustainability. Qiu et al. [126] synthesized gold nanoparticles containing β-cyclodextrin (β-CD@AuNPs) and prepared uniformly ordered β-CD@AuNP thin films through an assembly process. Subsequently, deep learning (DL) methods were employed for analysis. The study found that 1-hydroxypyrene concentrations as low as 0.05 µg/mL could be detected using the β-CD@AuNP thin films, with a relative standard deviation of 5.5%. Additionally, a classical deep learning network architecture, Convolutional Neural Network (CNN), was used to construct a predictive model, which was further optimized using a GA. The optimized CNN model, combined with the genetic algorithm, achieved root mean square errors of 0.9639 and 0.6327 for the training and prediction sets, respectively.

**Figure 9:**
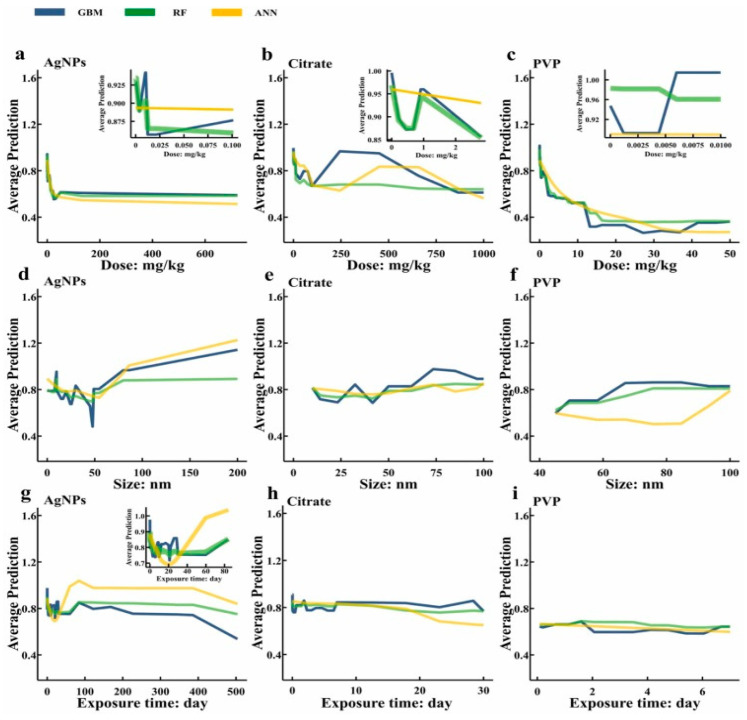
Partial dependence curves created for GBM, ANN, RF models: uncoated Ag NPs (**a**,**d**,**g**), citrate-Ag NPs (**b**,**e**,**h**), PVP-Ag NPs (**c**,**f**,**i**) [130].

**Ag NPs:** Ag NPs are prevalent in production, usage, and disposal, and are readily released into the environment. This is particularly true for Ag NPs used as pesticides and fertilizers, which can directly contaminate soil and negatively impact soil enzymes, organic matter, microorganisms, and ecological balance [127,128,129]. Enzyme activity serves as a critical indicator of soil microbial health, and employing ML to predict the effects of Ag NPs on soil enzyme activity can enhance the understanding of nanoparticle safety and sustainability in environmental contexts. Zhang et al. [130] compiled the literature on Ag NPs and soil enzyme experiments from the past decade, selecting 721 datasets to create a comprehensive database. They conducted an experiment using three ML models—ANN, GBM, and RF—to investigate the effects of dose, size, and exposure time of silver nanoparticles on soil enzyme activity. The results of the study are shown in Figure 9. The study revealed that the ANN optimized with GA (MAE = 0.1174) was most effective at simulating overall trends. The ANN model predicted that enzyme activity initially declined and then increased with increasing Ag NPs size. Partial dependence profile (PDP) analysis indicated that polyvinylpyrrolidone-coated Ag NPs display the importance of four factors affecting Ag NPs impact as follows: dose > type > size > exposure time. The RF model indicated that soil enzyme activity was most inhibited when experiments were conducted with doses of 0.01–1 mg/kg, particle sizes of 50–100 nm, and exposure times of 30–90 day. This study provided new insights into soil enzyme responses to Ag NPs and explored their ecotoxicity. Lin et al. [131] reviewed studies on Ag NPs and soil enzymes from 2010 to 2022, collecting 906 datasets for model development and analysis using the Web of Science database. The results of the study revealed that among the various regression models, the RF regression model yielded the most accurate predictions. Additionally, it was found that soil properties played a significant role in influencing the impact of Ag NPs on soil enzyme activity. Among these properties, pH had the greatest effect, with Ag NPs exerting a stronger influence on enzyme activity in acidic soils (pH < 5.5). Organic matter (OM) also had a notable effect, with a decrease in OM potentially reducing the ecotoxicity of Ag NPs. These findings contribute to predicting the global effects of Ag NPs on soil enzymes and identifying soil types that are more sensitive to Ag NPs on a global scale.

**Others:** With the rapid advancement of nanotechnology, engineered nanomaterials have been integrated into various production and consumer products. The widespread use of nanoparticle-containing products has heightened unprecedented concerns about the potential adverse effects of engineered nanoparticles (ENPs) on ecological health [132]. Wang et al. [133] assessed and predicted the toxicity of ENPs to aquatic organisms at the community or ecosystem level by collecting threshold concentration data for metal ENPs. They developed nine classification models and four regression models using eight supervised ML methods. These models were utilized for qualitative and quantitative predictions of HC5 (hazardous concentrations at which 5% of species are harmed) values. Summarizing the model prediction performance, all classification models achieved internal validation accuracies ranging from 71.4% to 100%, and regression models had R-squared values between 0.702 and 0.999, indicating strong performance. The regression models established in this study provide a solid methodological foundation for the rapid screening and evaluation of environmental risks associated with aquatic ecosystems, contributing to a better understanding of nanoparticle safety in environmental contexts. Zhang et al. [134] used ML -driven QSAR models to predict the ecological toxicity of engineered nanoparticles such as CuO NP, ZnO NP, and ZrO_2_ NP. They applied two ML techniques, namely SVM and neural networks (NN), and compared their predictive abilities against two component-based mixture models (independent action and concentration addition) for joint toxicity prediction. Among the 72 QSAR models developed using ML methods, two SVM-QSAR models and two NN-QSAR models demonstrated good performance. The NN-based QSAR model, which incorporated the molecular descriptors ΔH sf (enthalpy of formation of gaseous cations) and ΔH_(me+) (standard molar enthalpy of formation of metal oxides), exhibited the best predictive capability for the internal dataset (R^2^_text_ = 0.911, adjusted R^2^_test_ = 0.733, RMSE_test_ = 0.091, and MAE_test_ = 0.067). This approach provides a methodological and theoretical basis for assessing the ecological risks of ENP mixtures and offers pathways to deepen the understanding of nanoparticle toxicity and sustainability in ecological environments.

### 5.3. Summary

In the field of environmental engineering, both traditional methods and ML approaches are crucial for evaluating the safety and sustainability of metallic nanoparticles. This section reviews their effectiveness in predicting the biosafety and sustainability of metal nanoparticles in environmental applications. Table 3 contrasts traditional and ML approaches in evaluating the safety and sustainability of metallic nanomaterials in environmental engineering applications.

## 6. Others

In recent years, the application range of MNPs has expanded significantly. Beyond their well-known uses in medicine, energy, and environmental fields, MNPs are increasingly utilized in areas such as food, cosmetics, and agriculture [135,136]. In the food industry, MNPs are employed as food additives to enhance color, texture, and flavor, and are also used for food safety detection. In cosmetics, MNPs are used to improve product performance and stability. In agriculture, nano-formulated pesticides, fertilizers, and other agrochemicals are being developed to increase crop yields and improve soil quality. However, these applications have also raised significant concerns regarding potential health and environmental risks. Therefore, comprehensive research and thorough assessment of the safety of MNPs are crucial.

### 6.1. Traditional Method in Other Fields

**Figure 10:**
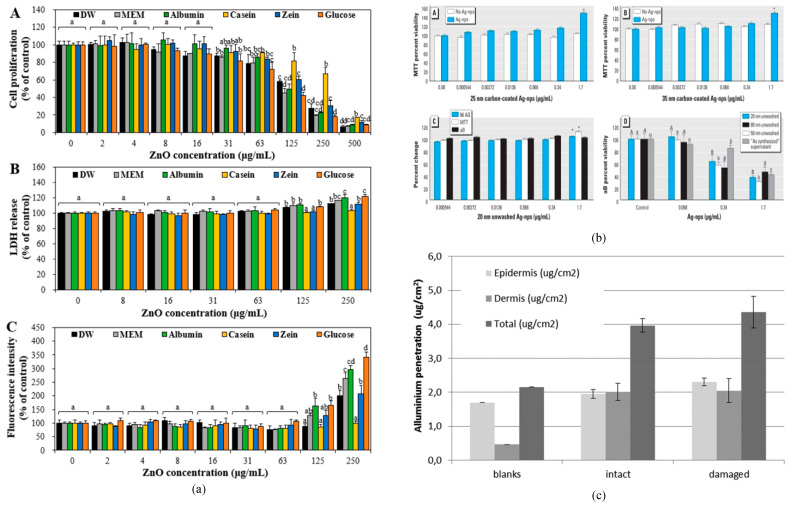
Potential toxicity of metal nanoparticles in food and cosmetics. (**a**) The interaction of ZnO NPs with food proteins (albumin, casein, zein, etc.) affects various factors of the food proteins. (**b**) Evaluating the activity of Ag NPs Using MTT, 96AQ, and aB methods, respectively. (**c**) Distribution of aluminum oxide nanoparticles within human skin [139,142,143].

**Food field:** In recent years, the manufacturing technology for food additives has advanced rapidly, leading to a greater variety of additives. While this development brings convenience to society, it also presents new challenges [137]. Nanomaterials used as food additives may pose potential health risks, making it essential to assess their safety to ensure that foods containing these additives do not harm human health. Due to their nutritional value, zinc oxide (ZnO) NPs are used as zinc supplements [138]. ZnO NPs have the potential to interact with food matrices, which could lead to varying biological responses. Therefore, it is important to elucidate the toxicity of ZnO NPs in the food industry. Jung et al. [139] evaluated ZnO NPs interactions with food proteins (albumin, casein, zein) via microscopy and in vivo oral experiments. As depicted in Figure 10a, ZnO NPs reduced albumin’s hydrodynamic diameter but enhanced cytotoxicity, cellular uptake, and intestinal transport, while preserving its structural stability and pharmacokinetics. A 14-day mouse study revealed low acute oral toxicity. Although ZnO NP–protein interactions modulate in vitro responses, chronic toxicity assessment requires longer-term studies. Current research emphasizes single nanomaterial toxicity, neglecting the nanotoxicology of composites. Nanomaterials in food additives may react with food elements, forming toxic compounds, emphasizing the need for toxicity studies on composite systems. Wang et al. [138] assessed the combined toxicity of ZnO nanoparticles and vitamin C by evaluating their dose–response relationships within cellular environments. They found that exposure to ZnO NPs at concentrations below 15 mg/L or vitamin C below 300 mg/L did not cause significant cytotoxicity in gastric epithelial cell lines (GES-1) or neural stem cells (NSCs). However, when both ZnO NPs and vitamin C were introduced together, cell viability decreased sharply, indicating that vitamin C significantly increased the cytotoxicity of ZnO NPs compared to ZnO NPs alone. This study addresses a gap in the research on the safety of composite nanomaterials.

**Cosmetic field:** Due to the rapid growth of the cosmetics industry in recent years, a variety of nanomaterials with unique physicochemical properties are now being incorporated into cosmetic formulations. Many of the cosmetics we use daily are produced with the aid of nanotechnology. The addition of silver and gold nanoparticles can also enhance the antimicrobial and healing properties of certain cosmetic formulations. To accurately assess the benefit–risk balance of these products, it is essential to estimate the potential hazards associated with NP exposure levels [140]. Ag NPs are among the most commonly used in cosmetics, accounting for approximately 12% of all nanomaterials used in cosmetic products [141]. Samberg et al. [142] investigated the potential cytotoxicity of Ag NPs in human epidermal keratinocytes (HEK) using MTT and 96AQ assays, as well as direct microscopic observation. They also assessed the inflammation and permeation potential of Ag NPs in pig skin. The study encompassed eight Ag NP types—unwashed/uncoated (20, 50, 80 nm), washed/uncoated (20, 50, 80 nm), and carbon-coated (25, 35 nm)—applied to skin for 14 days. HEK viability was evaluated using MTT, alamarBlue, and 96AQ assays, while pro-inflammatory cytokines (IL-1β, IL-6, IL-8, IL-10, TNF-α) were measured. Unwashed Ag NPs significantly reduced HEK viability in a dose-dependent manner. Pig skin showed no macroscopic irritation but microscopic inflammation and Ag NPs presence. Washed or carbon-coated Ag NPs were non-toxic in cosmetic applications. This research offers valuable insights into Ag NP toxicity and permeability over 14 days, contributing to MNPs’ risk assessment. Mauro et al. [143] employed histological staining and other methodologies to observe the distribution of aluminum oxide nanoparticles (Al_2_O_3_ NPs) with diameters ranging from 30 to 60 nm within human skin, as a means to study their percutaneous absorption. The study used Franz cells to investigate the permeability of 30–60 nm Al_2_O_3_ NPs dispersed in synthetic sweat (20 g/L) on excised human skin. The skin permeability was found to be 0.0028% for abraded skin and 0.0011% for intact skin. These results strongly suggest that Al_2_O_3_ NPs are sufficiently safe, as the percentages of Al elements detected are consistent with the natural content of aluminum in the human body.

**Agriculture field:** As metal nanomaterials have developed in the agricultural field, these materials can be used as fertilizers, pesticides, herbicides, sensors, and quality enhancers, enabling the production of more food at lower costs, energy, and waste. However, many questions regarding the risks of these methods in food production remain unanswered. Therefore, it is particularly important to assess the potential hazards of NPs to crops and their processed products [144]. Currently, a significant knowledge gap in nanotechnology is the lack of standardized protocols for testing the effects of metal nanoparticles on soil microorganisms. Chai et al. [145] employed a combination of thermometabolic monitoring, functional bacterial monitoring, and enzyme activity monitoring to detect the ecotoxicity of nanoparticles in agricultural soil. Agricultural soil was exposed to ZnO, SiO_2_, TiO_2_, and CeO_2_ nanoparticles at a concentration of 1 mg g^−1^, and soil microbial activity in the presence and absence of these four metal oxide nanoparticles was studied, as shown in Figure 10c. The study indicated that superoxides and reactive oxygen species produced by the nanoparticles might reduce microbial biomass and activity. TiO_2_ and ZnO nanoparticles decreased substrate-induced respiration in grassland soil. This research thoroughly demonstrated the impact of metal nanoparticles on agricultural soil, significantly aiding future safety assessments of metal oxide nanoparticles. To investigate the effects of NPs on plants and soil microorganisms, Barrena et al. [146] conducted standard toxicity tests to determine the toxicity of these nanoparticles, performing germination (cucumber and lettuce), bioluminescence (*Vibrio fischeri*), and anaerobic toxicity tests. Germination tests were conducted on Au, Ag, and Fe_3_O_4_ at NP doses of 62, 100, and 116 μg/mL, respectively. Bioluminescence tests were conducted on Au, Ag, and Fe_3_O_4_ at doses of 28, 45, and 52 μg/mL, respectively. Finally, anaerobic tests were conducted on Au, Ag, and Fe_3_O_4_ at NP doses of 10, 16, and 18 μg/mL, respectively. The results indicated that at the tested concentrations, the gold, silver, and Fe_3_O_4_ nanoparticles exhibited low or zero toxicity. This study emphasized the need for a deeper understanding of the interactions between nanoinorganic materials and the environment before their large-scale agricultural application.

### 6.2. Machine Learning Methods in Other Fields

**Figure 11:**
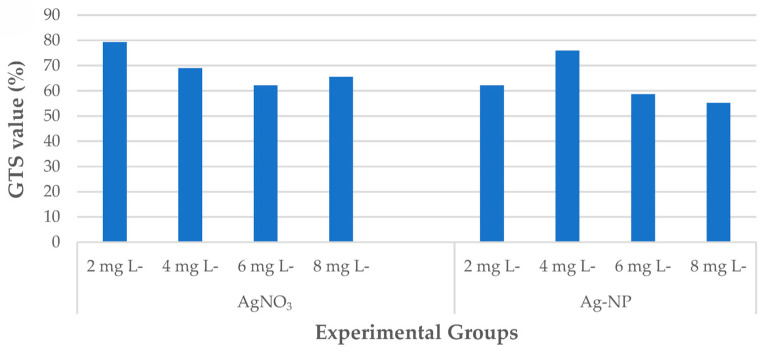
GTS values, estimated with different experimental groups [148].

**Food filed:** With advancements in nanotechnology, metal nanoparticles are increasingly employed across various industries due to their advantageous physicochemical properties. However, their widespread use has led to significant accumulation in soil environments, which not only diminishes crop yields at higher concentrations but also poses risks to food safety and security through contamination of the food supply [147]. Türkoğlu et al. [148] explored the effects of AgNO_3_ and Ag-NPs on wheat regeneration and DNA methylation using ML methods, including SVM, RF, extreme gradient boosting (XGBoost), k-nearest neighbor classifier (KNN), and Gaussian process classifier (GP). Ag-NPs at 2 mg L^−1^ yielded the highest callus induction rate, while 8 mg L^−1^ AgNO_3_ optimized regeneration efficiency. AgNO_3_ at 2 mg L^−1^ achieved the highest genomic template stability (79.3%), as shown in Figure 11. Elevated Ag NO_3_ levels increased DNA hypermethylation, and higher Ag-NP levels led to hypomethylation. The study highlights combining these ML models for better wheat in vitro characterization and notes the safety concerns regarding metal oxide nanoparticles in food additives. Sayed et al. [149] proposed a ML model to predict the potential toxicity of metal oxide nanoparticles, utilizing a dataset of 79 descriptors for 24 metal oxide nanoparticles (Me_x_O_y_ NPs) and their physicochemical and structural characteristics. The experimental results demonstrated that the feature selection algorithm based on the Sine Tree-Seed Algorithm (EBSTSA) was both reliable and robust, performing well on 23 benchmark datasets from the UCI ML Library. The EBSTSA efficiently identified relevant descriptors for nanoparticles. Additionally, the proposed ML toxicity prediction model exhibited an average error rate of 1.02%, specificity of 100%, sensitivity of 98.87%, and an F1 score of 99.47%. This study presents a validated and user-friendly ML model for predicting the cytotoxicity of metal oxide nanoparticles, enhancing risk assessment and informed decision making in industrial and biological applications.

**Cosmetic field:** ZnO NPs are widely used in foods, pharmaceuticals, sunscreens, and cosmetics as active ingredients or color additives. While no strong evidence directly links ZnO NPs to serious health risks in everyday use, the scientific data on their safety is inconclusive and often lacks in vivo validation [150]. To address the controversy stemming from inconsistent results in previous in vitro toxicity studies of ZnO NPs, Bilgi et al. [151] conducted a synthesis of over 25 independent studies. Using one-way analysis of variance (ANOVA) and categorical regression tree (CART) algorithms, they identified both endogenous and exogenous factors affecting the cytotoxic potential of ZnO NPs. Their findings highlighted that particle size significantly influences cytotoxicity, with sizes below 10 nm showing enhanced cytotoxic effects. The study also indicated that exposure durations of 12 h or more consistently resulted in cytotoxic responses, irrespective of NP concentration. This meta-analytical approach is crucial for leveraging accumulated data in nanosafety studies and advancing research over time. Titanium dioxide nanoparticles (TiO_2_ NPs) are widely used in cosmetics like makeup and sunscreens for their brightness, opacity, and antimicrobial properties. However, their extensive use raises concerns about potential health risks, highlighting the need for urgent risk assessment under various exposure conditions [152]. Leroux et al. [153] studied the effects of TiO_2_ nanoparticles on NR8383 cells (alveolar macrophage precursors) and Fischer 344 rats. Using WST-1 and LDH assays, they found low cytotoxicity in vitro. Despite no observed toxicity, both exposure conditions triggered 18 common differentially expressed genes (DEGs) linked to cell proliferation, apoptosis, and inflammation. Notably, CCl_4_, Osm, CCl_7_, and BCl_3_ genes were up-regulated, indicating these as potential early biomarkers of TiO_2_ NP exposure. In vivo, rats inhaled 10 mg/m^3^ nanostructured aerosols for 6 h daily over 4 weeks, with similar gene expression changes observed.

**Agriculture field**: Nanotechnology is increasingly advancing and being applied in agriculture. However, nano-agriculture is fraught with uncertainties, necessitating the development of intelligent methods to assess these uncertainties, promote nano-agriculture, and mitigate risks to plant growth. Deng et al. [154] developed a database of 1174 datasets and used 13 Random Forest models (R^2^ > 0.8) to predict plant responses to nanoparticles. They found that plant responses were mainly influenced by factors like exposure dose, duration, plant age, NP size, and zeta potential. The study also highlighted regional challenges in nano-agriculture, such as Fe_2_O_3_ NPs potentially inhibiting bean growth in Europe due to low night-time temperatures. The research suggests that rising temperatures could reduce NP-induced stress in crops, offering insights into the future of nano-agriculture through ML. Xu et al. [155] integrated raw high-throughput sequencing (HTS) datasets from 365 soil samples to investigate the ecological effects of NPs on soil microbial communities using metadata analysis and ML methods. Metadata analysis revealed that nanoparticle treatments did not significantly impact the α-diversity of microbial communities but notably altered β-diversity. Additionally, nanoparticle treatments were found to reduce the biosynthetic capacity for cofactors, carriers, and vitamins, while enhancing the degradation of aromatic compounds and amino acids. Their proposed RF model demonstrated potential for identifying critical NP properties affecting soil microbial community stability, with high predictive performance (mostly R² > 70%). This study examines the relationship between nanoparticle properties and soil microbial community hazards from a macroscopic perspective, offering guidance for the industrial, agricultural, and manufacturing applications of NPs to minimize environmental risks.

### 6.3. Summary

This section examines the biosafety and sustainability of metal nanoparticles in other fields and provides an overview of the application of traditional and ML methods in this research, respectively. Table 4 contrasts traditional and ML approaches in evaluating the safety and sustainability of metallic nanomaterials in other fields.

## 7. Discussion

Analyzing the toxicity and sustainability of metal nanoparticles is pivotal in ensuring their safe application. The traditional methods and ML approaches offer distinct perspectives for a comprehensive evaluation of the risks and environmental properties associated with these nanoparticles. Traditional methods and ML algorithms have their own advantages and disadvantages, which will be discussed in this section from the perspective of the two methods themselves as well as in comparison.

### 7.1. Analysis of Traditional Methods

**Figure 12:**
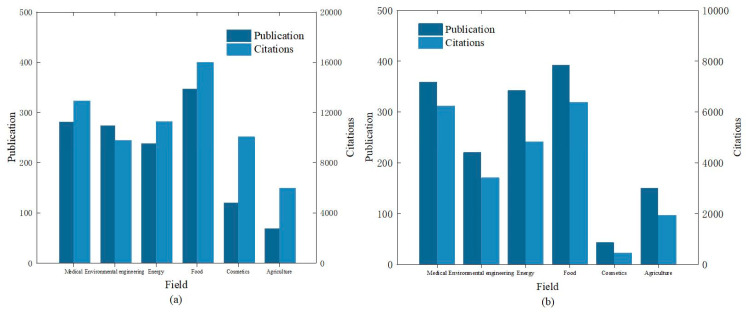
Number of publications and citation frequency of articles in web of Science on traditional methods for detecting toxicity of metal nanomaterials in different fields: (**a**) by 2019; (**b**) 2020–2024.

In the bar charts of Figure 12a,b, different colors represent the number of publications and citations for traditional methods of detecting metal nanomaterials (such as gold and silver nanoparticles), and data from six different fields were recorded. The charts illustrate that the fields of medicine and food place significant emphasis on toxicological research of metal nanomaterials, while other fields, such as cosmetics, receive relatively less attention. The charts effectively compare research data from the past five years and more than five years ago using traditional methods to detect the toxicity of metal nanomaterials, reflecting a declining trend in the importance of traditional methods in these fields. In past studies, cell models combined with metal nanoparticles were frequently used for toxicity research, providing a direct view of the hazards posed by metal nanomaterials to cells. However, this method still has many uncertainties, highlighting the need for substantial improvements in the sustainability and safety of metal nanomaterials.

As metal nanomaterials are widely applied in the environment and daily life, traditional methods have seen diminishing use in the fields of energy and environmental engineering. This is due to the numerous limitations of traditional methods in effectively detecting the safety risks of metal nanomaterials in these areas. Conversely, traditional methods continue to gain more attention in fields such as food and medicine. This increased focus is not only because these fields are closely related to human life but also because traditional methods can more intuitively reveal the hazards of metal nanomaterials in these applications. With the development of AI technology, ML and AI methods are being increasingly employed to detect the safety of metal nanomaterials with greater accuracy compared to traditional methods. These advanced methods address the issues of the substantial time and labor consumption required for material testing, enabling high-throughput screening and big data analysis, thereby accelerating the design and application of metal nanomaterials.

### 7.2. Analysis of Machine Learning Methods

**Figure 13:**
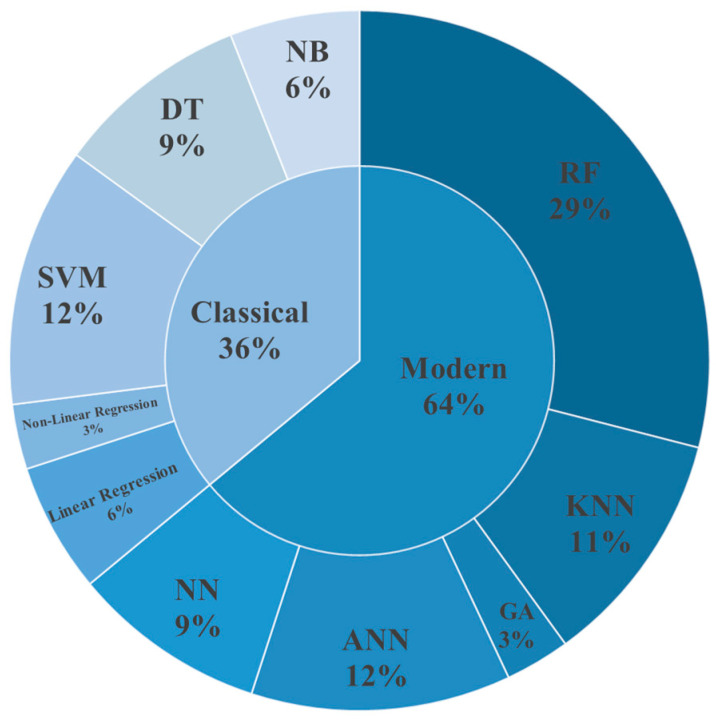
Distribution of ML modeling methods.

Differences exist in studies concerning the use of ML models to predict the toxicity of metal nanomaterials (such as Au NPs and Ag NPs) across various fields. This paper summarizes and categorizes the articles discussing the safety of metal nanomaterials through ML methods. ML approaches for predicting the toxicity of metal nanomaterials have garnered significant attention and research within the medical field. In contrast, research related to the toxicity of metal nanomaterials in the food, cosmetic, and agricultural sectors remains limited, likely due to the exploratory nature of metal nanomaterial applications in these areas and the incomplete experimental data available. Figure 13 illustrates the proportion of traditional versus modern methods applied in ML models and their classification. It is evident that modern methods are more prevalent than traditional ones. This preference is attributed to the fact that traditional algorithms may be less effective and exhibit higher error rates in domains with limited data. Modern methods, such as Random Forests, Artificial Neural Networks, and k-nearest neighbors, are increasingly employed to enhance toxicity prediction by integrating multiple ML models. This approach reduces the bias and variance associated with individual models, leading to more reliable predictions.

### 7.3. Comparative Analysis of Methods

Traditional methods for measuring toxicity typically involve in vitro and in vivo experiments to directly observe biological responses. While these methods are highly credible and interpretable, they are time-consuming, costly, and require significant amounts of experimental materials and reagents. Additionally, traditional methods demand strict experimental environments, complex operations, and specialized personnel. In contrast, ML methods assess toxicity using big data and algorithmic models. Although the initial stage requires substantial high-quality data for training, which can be costly, once established, the model’s running costs are low. ML methods can process and analyze large datasets quickly, yielding rapid results without producing experimental waste, thus offering environmental benefits. Furthermore, ML methods excel in analyzing complex data patterns and predicting unknown toxicity, enhancing prediction accuracy and reliability, and are suitable for high-throughput screening and large-scale toxicity assessment. In conclusion, traditional methods remain important due to their directness and reliability, while ML methods hold broad prospects for future toxicity detection and prediction due to their efficiency and scalability. Combining the strengths of both approaches to establish a comprehensive toxicity evaluation system will facilitate a more thorough and accurate assessment of the safety of metal nanoparticles.

Combining traditional and ML methods leverages the strengths of both approaches. Traditional methods provide reliable experimental data and direct toxicity observations, which can serve as training and validation sets for ML models, enhancing their accuracy and credibility. The high quality and authenticity of experimental data lay a solid foundation for ML algorithms, enabling more precise toxicity predictions for unknown samples. ML methods excel at processing large datasets, quickly analyzing and uncovering underlying patterns and relationships. This capability allows ML methods to extract key features from extensive experimental data and perform rapid predictions and screenings, significantly improving toxicity assessment efficiency. By integrating accurate experimental results from traditional methods, ML can optimize experimental design and reduce unnecessary experiments, saving costs and time. Additionally, combining these methods facilitates high-throughput screening and personalized assessments. Traditional methods can be employed for detailed studies of toxicity mechanisms, while ML can conduct large-scale toxicity predictions and risk assessments, allowing for extensive screening and in-depth analysis in specific cases. This comprehensive assessment system can be tailored to various research needs, supporting everything from basic research to application development. In conclusion, integrating traditional and ML methods provides a powerful and flexible tool for assessing the toxicity of metal nanoparticles, enabling a more comprehensive understanding of their safety and promoting their safe application across different fields.

## 8. Outlooks

Metal nanoparticles have a wide range of applications in medicine, energy, and environmental engineering. However, their potential biosafety and environmental impact have gradually attracted attention. To address these challenges, two types of toxicity detection methods have been developed: traditional detection methods and ML methods. With ongoing technological advancements and in-depth research, the approaches for detecting the toxicity of metal nanoparticles will continue to improve and refine. Future research on the safety of metal nanoparticles should focus on in-depth mechanism studies, interdisciplinary collaboration, and the development of more efficient and environmentally friendly detection technologies. Specific research directions are listed below.

(1) Metallic nanomaterials are extensively utilized in fields such as medicine and food due to their unique physical and chemical properties. Traditional methods, which are well-established techniques, remain among the most commonly used approaches for predicting their toxicity. These methods typically involve using cellular models combined with metal nanoparticles to detect toxicity. However, such experiments are time-consuming and labor-intensive. With the rapid advancement of science and technology, future research should focus on improving the accuracy and simplicity of these models, enhancing data processing capabilities and making significant breakthroughs in the principles and techniques. These improvements will advance traditional methods in the field of toxicity prediction, thereby better safeguarding the safe application of metallic nanomaterials in areas such as medicine and environmental engineering.

(2) Looking ahead, with the maturity of ML technology and the increasing availability of computational resources, the field of predicting the toxicity of metal nanoparticles using ML algorithms will see significant advancements. We anticipate the development of more refined and efficient models capable of accurately capturing the toxicity variations in metal nanoparticles under different environmental conditions, such as pH value, temperature, and surface modifications. Additionally, the creation of real-time dynamic prediction models will become a crucial focus, aiming to enable the immediate monitoring and assessment of nanoparticles’ behaviors and toxic effects in living organisms. This is particularly important for nanomedicine research and development, environmental governance, and occupational health protection. Furthermore, in the era of big data, efficiently integrating and utilizing massive data resources, optimizing ML algorithms, and improving prediction accuracy and generalization capabilities will be ongoing challenges that need to be addressed in this field.

(3) Traditional methods and ML algorithms each have their own advantages and limitations in detecting the toxicity of metallic nanomaterials, and they should not be viewed as mutually exclusive. Combining these approaches can leverage the strengths of both: traditional methods offer experimental validation and mechanistic insights, while ML algorithms excel in data processing, pattern recognition, and predictive capabilities. This synergy can enhance the accuracy and scientific rigor of toxicity predictions. As technology continues to advance, we anticipate the development of more innovative fusion strategies. These might include integrating complex physicochemical parameters, biological reaction kinetic models, and advanced ML architectures to create more detailed and comprehensive prediction models. Such models will not only better simulate the behavior of nanoparticles in biological environments but also effectively predict their toxic effects under various exposure conditions, providing more reliable data for the safety assessment of nanomaterials.

(4) Interdisciplinary collaboration and data sharing are essential for advancing this field. By fostering exchanges and cooperation among materials science, toxicology, computer science, and biomedicine, we can develop a more comprehensive toxicity prediction system and expedite the translation and application of research findings. Establishing unified data standards and a shared platform will facilitate the convergence and integration of research data, ensuring the continuous optimization and generalization of ML models.

## Figures and Tables

**Table 1:** Traditional and ML approaches to detect metal nanomaterials toxicity in medical field.

Methods	Test Materials	Test Object	Experimental Method	Time and Dose	Remarks	Reference
Traditional methods	Au NPs (1.4 nm and 18 nm)	WI-38 and A549	MTT	10 μg/mL for 72 h	CuO NPs can inhibit the proliferation and viability of normal and lung cancer cells.	Semmler-Behnke et al. [51], 2020
	Ag NPs	In vitro rat liver derived cell line	MTT	10–50 μg/mL for 24 h	Ag NPs can lead to a significant decrease in GSH levels, a decrease in mitochondrial membrane potential, and an increase in ROS levels in rat liver-derived cells.	Hussain et al. [58], 2005
	Cu NPs	PC12 cells	MTT	1, 10, 30, and 100 μg/mL for 24 h	The intracellular accumulation of Ros gradually increased with increasing concentrations of Cu NPs, and oxidative stress was a key mechanism of apoptosis induced by nano-Cu in PC12 cells.	Xu et al. [68], 2012
ML methods	Au NPs	Human monocyte-derived macrophages	RF, SVM, NB, DT, ANN	Not specified	Among the five models, the RF model exhibited the best performance, achieving the highest predictive accuracy, F-measure, sensitivity, and specificity.	Amadi et al. [69], 2024
	Ag NPs	Characteristics such as particle size, Zeta potential, cell type, etc.	DT, RF		The Random Forest (RF) model achieved the highest accuracy of 0.825 and a predictive power of 0.904.	Liu et al. [75], 2021

**Table 2:** Traditional and ML approaches to detect metallic nanomaterials safety and sustainability in energy field.

Methods	Test Materials	Test Object	Experimental Method	Time and Dose	Remarks	Reference
Traditional methods	Ag NPs	Liver cells and fibroblasts	XTT	25 and 100 μg/mL for 24 h	Although Ag NPs enter eukaryotic cells, the cell’s antioxidant machinery protects the cells from possible oxidative damage.	Arora et al. [85], 2009
	Pt NPs	EVT	ICP-MS	12.5 and 50 μg/mL for 24 h	A novel protective mechanism against NPT cytotoxicity in human trophoblast cells was discovered.	Nakashima et al. [90], 2019
	Ir-192 nanoparticles	Lymphocytes	LA-ICP-MS	17, 140, 430, and 690 μg/m^3^ for 1 month	The human body could be fatally harmed by bare Ir-192 NPs, which were toxic nanoparticles.	Buckley et al. [98], 2017
ML methods	Au NPs	Thermodynamic stability	LDA ML-FF, rPBE ML-FF	Not specified	Provide a data-driven definition of liquid atomic arrangements in the inner and surface regions of a nanoparticle and employ it to show that melting initiates at the outer layers.	Zeni et al. [102], 2021
	Ag NPs	Dynamic viscosity	ANN		The ANN can predict the viscosity of Ag/ethylene glycol nanofluid with good precision compared to the correlation method.	Toghraie et al. [106], 2019

**Table 3:** Traditional and ML approaches to detect metallic nanomaterials safety and sustainability in environmental engineering field.

Methods	Test Materials	Test Subject	Experimental Method	Time and Dose	Remarks	Reference
Traditional methods	Cu NPs	Juvenile rainbow trout	MTT	11, 300, 1000 µg Cu·g^−1^ for 28 days	In fish exposed to copper, apoptosis, mitosis, and eosinophilic granulosa cells were more noticeable.	Kamunde et al. [119], 2001
	Ag NPs	Aquatic plants	GF-AAS	2.5 mg Ag L^−1^ for 28 days	All three silver treatments are toxic to aquatic plants and lead to a large release of dissolved organic carbon and chlorine after exposure.	Colman et al. [122], 2014
ML methods	Au NPs	Ecotoxicity in the aquatic environment	RF, SVM, ANN	Not specified	Exposure duration, illumination, primary size, and hydrodynamic diameter were the main factors affecting the ecotoxicity of metallic nanomaterials to a variety of aquatic organisms.	Zhou et al. [125], 2023
	Ag NPs	Soil enzymes	DT, KNN		Soil properties played a pivotal role in determining AgNPs’ effect on soil enzymes.	Lin et al. [131], 2024

**Table 4:** Traditional and ML approaches to detect metallic nanomaterials safety and sustainability in other fields.

Methods	Test Materials	Test Subject	Experimental Method	Time and Dose	Remarks	Reference
Traditional methods	ZnO NPs	Female rats	Oral gavage	100 mg/mL for 14 days	Zn O NPs have low oral toxicity in vivo. The interactions between Zn O NPs and food proteins modulate in vitro biological responses, but do not affect in vivo acute oral toxicity.	Jung et al. [139], 2021
	Ag NPs	Human epidermal keratinocytes	96AQ, MTT	Skin was dosed topically for 14 consecutive days.	Microscopic and ultrastructural observations showed areas of focal inflammation and localization of Ag-NPs on the surface and in the upper stratum corneum layers of the skin.	Samberg et al. [142], 2009
ML methods	Ag NPs	Wheat regeneration, callus induction, and DNA methylation	RF, SVM, KNN	Not specified	The highest values for callus induction (CI%) and embryogenic callus induction (EC%) occurred at a concentration of 2 mg L^−1^ of Ag-NPs. Additionally, the regeneration efficiency (RE) parameter reached its peak at a concentration of 8 mg L^−1^ of AgNO_3_.	Türkoğlu et al. [148], 2023
	ZnO NPs	In vivo	ANOVA, CART		Particle size was found to have the most significant impact on the cytotoxic potential of ZnO NPs, with 10 nm identified as a critical diameter below which cytotoxic effects were elevated.	Bilgi et al. [151], 2024
